# Bacteriological profile and antimicrobial susceptibility patterns of urine culture isolates from patients in Ndjamena, Chad

**DOI:** 10.11604/pamj.2017.28.258.11197

**Published:** 2017-11-23

**Authors:** Michel Kengne, Amon Todjimbaide Dounia, Julius Mbekem Nwobegahay

**Affiliations:** 1 Department of Medical Microbiology and Immunology, School of Health Sciences-Catholic University of Central Africa, Yaoundé, Cameroon; 2 Ndjamena General Hospital of National Reference, Chad; 3 Yaoundé Military Hospital, Cameroon

**Keywords:** Bacteriological profile, antimicrobial susceptibility patterns, urine culture isolates

## Abstract

**Introduction:**

Bacteriological profile and antimicrobial susceptibility patterns of urine culture isolates were determined among patients in the Ndjamena General Hospital, a National Reference centre.

**Methods:**

A cross-sectional study was carried out from July to November 2014. Six hundred and sixty patients were enrolled, to whom a cytobacteriological examination of urine was prescribed. Urine was collected and cultured. Bacterial identification and antimicrobial susceptibility patterns were performed using Vitek 2 compact automated system.

**Results:**

216 isolates were recovered from patients (age range: 10-90 years). *E. coli* was the pathogen frequently cultured 128 (59.3%) followed by *K. pneumonia* 28 (13.0%). Bacteriuria was more present in inpatients (70.4%) compared to outpatients (29.6%). High antibiotic-resistance rate (> 60%) of the total isolates was observed with ampicillin, ciprofloxacin and cephalosporins. Imipeneme (94.9%) displayed satisfactory activity against bacteria isolates. ESBLs phenotype was present in 68/105 (64.7%) of betalactamine resistant isolates. AAC(3)-I and AAC(6’)-I enzymes were found respectively in 16/36 (44.4%) and 20/36 (55.6%) of aminoglycosides resistant isolates. Resistance of isolates to quinolones was mainly due to an association of target modification (gyrA and parC), porin reduction and/or efflux mechanisms and was present in 107/213 (49%) of quinolones resistant isolates.

**Conclusion:**

*E. coli* is the predominant uropathogen isolated in our setting and there are antibiotic-resistant uropathogens among the studied population. Therefore, routine surveillance of bacterial uropathogens to common used antibiotics must be a continuous process so as to provide physicians with up to date information about the local data of antimicrobial resistance.

## Introduction

Urinary tract infections (UTIs) are the third most common infection in human with an estimated annual global incidence of at least 250 million in developing country [1-3]. Different microorganisms can cause UTIs, *E. coli*which accounts approximately for 75% of isolates is the most common pathogen isolated from community and hospital acquired UTIs [4]. Other uropathogens such as *Klebsiella spp.* and *S. saprophyticus* have also been frequently isolated [5, 6]. The management of UTIs is usually empirical, based on the predictable spectrum of etiologic agents and their susceptibility patterns. Due to the emergence of antimicrobial resistance among uropathogens, the effectiveness of empirical therapy has been affected [7]. In Chad, antimicrobial susceptibility among pathogens involved in UTIs is poorly investigated. Therefore, it is important to obtain information on local antimicrobial resistance rates to provide adequate treatment to community and hospital acquired UTIs in this country. In this study, we investigated the bacteriological profile and the antimicrobial susceptibility patterns of urine culture isolates among patients in Ndjamena General Hospital of National Reference.

## Methods

**Patients:** The study participants were both inpatients and outpatients attending the Ndjamena General Hospital of National Reference from July to November 2014. Six hundred and sixty patients to whom a cytobacteriological examination of urine was prescribed and who had not received antimicrobials within the previous fifteen days were eligible for inclusion.

**Bacterial isolation, identification procedures and antimicrobial susceptibility testing:** A freshly midstream morning urine sample of 2-5ml was collected in sterile container from the selected patients. Aseptic measures were maintained during samples collection. Immediately after sampling, urine was spread on Eosin Methylene Blue agar media and incubated at 37°C for 24h. A growth of > 105 CFU/ml of one type of organism was considered as significant bacteriuria. Identification of the pathogens and antimicrobial testing were performed using Vitek 2 compact automated system (bioMérieux, Marcy l’étoile, France) according to the manufacturer’s instructions.

The results were recorded as susceptible (S), susceptible dose dependent (SDD) and resistant (R). The following antibiotics purchased from bio-Mérieux (France) were used: amoxicillin/ clavulanic acid (20/10μg), ampicillin (10μg), oxacillin (5μg), ceftriaxone (30μg), ceftazidime (30μg), cefotaxime (30μg), aztreonam (30μg), imipenem (10μg), gentamycin (15μg), amikacin (30μg), nalidixic acid (30 μg), ciprofloxacin (5μg), ofloxacin (5μg). The reference strain used as quality control was *E. coli* (ATCC 25922).

**Data analysis:** Data were entered and analyzed using SPSS version 12.0 for windows (SPSS, Inc., Chicago, IL). Discrete variables were expressed as frequencies and percentages.

**Ethics:** Permission to collect samples was obtained from the Ndjamena General Hospital of National Reference. Informed consent was obtained from all participants ≥ 18 years, while assent consent was obtained from participant teenagers via a proxy.

## Results

From July to November 2014 (05 months study period), 660 urine samples were obtained from patients (age range: 10-90 years) and analyzed. 216 (32.7%) samples had significant bacteriuria among which 94 (43.5%) positive samples were from women and 122 (56.5%) were from men (sex ratio M/F: 1.29). Bacteriuria was more present in inpatients 152/216 (70.4%) compared to outpatients 64/216 (29.6%). The most frequent pathogen was *E. coli* 128 (59.2%), followed by *K. pneumonia* 28 (13.0%) and *E. cloacae* 11 (5.1%) which altogether accounted for 77.3% of the total isolates (Table 1). Bacterial uropathogen isolates from patients revealed the presence of high level of single and multiple antimicrobial resistances against commonly prescribed drugs. The total isolates displayed a resistance rate of > 60% to the antibiotics used excepted imipéneme, amikacin, aztreonam and gentamicin for which the resistance rates were 2.7%, 19.4%, 30.5% and 49.5% respectively. *E.coli* which is the predominant cause of UTIs in this study was susceptible to imipeneme 96.1%, aztreonam 67.1%, amikacin 62.5% and gentamycin 50%. As *E. coli*, *K. pneumonia* and *E. cloacae* were the most important uropathogens isolated and their susceptibility rates are displayed in Table 2.

**Table 1:** Distribution of uropathogens in patients

Organism	Inpatients n° of isolates (%)	Outpatients n° of isolates (%)
*Escherichia coli*	83 (38.4)	45 (20.8)
*Klebsiella pneumoniae*	24 (11.2)	4 (1.8)
*Enterobacter cloacae*	11 (5.1)	0 (0)
*Proteus mirabilis*	4 (1.8)	2 (0.9)
*Providencia stuartii*	1 (0.4)	3 (1.4)
*Klebsiella oxytica*	4 (1.8)	0 (0)
*Shigella spp*	2 (0.9)	2 (0.9)
*Serratia odorifera*	4 (1.8)	0 (0)
*Morexella lacunata*	3 (1.4)	0 (0)
*others*	16 (7.4)	8 (3.7)
*Total 216 (100)*	152 (70.4)	64 (29.6)

**Table 2:** Percentage of resistance of isolates to antibiotics

Antibiotics Isolates		All isolates(n= 216)	E. coli(n= 128)	K. pneumonia(n= 28)	E.cloacae (n= 11)
Betalactamines	Amoxi+Acclavulanique	83.3	80.5	100	90.9
	Ampicillin	92.6	91.4	100	100
	Oxacillin	92.6	93.7	100	90.9
	Ceftriaxone	67.6	62.5	85.7	90.9
	Ceftazidime	61.1	54.7	85.7	81.8
	Cefotaxime	67.1	59.4	96.4	90.9
	Aztreonam	30.5	25.0	42.8	36.4
	Imipenem	2.7	1.0	0	0
Aminoglycosides	Gentamicin	49.5	47.6	46.4	72.7
	Amikacin	19.4	13.3	3.6	45.4
Quinolones	Ciprofloxacin	72.7	70.3	89.3	63.6
	Ofloxacin	83.3	78.9	100	90.9
	Nalidixic acid	80.1	80.5	89.3	90.9

Furthermore, the remainder of this report will consider only these organisms. Betalactame-type enzymes production which determines variable phenotypes of betalactamines resistance was observed among the isolates. Extended spectrum betalactamases (ESBLs) that hydrolyze extended-spectrum cephalosporins (cefotaxime, ceftriaxone, ceftazidime) and monobactams (aztreonam) were present in 68/105 (64.7%) of betalactamines resistant isolates followed by cephalosporinase overproducing phenotype 27/105 (25.7%). Aminoglycosides resistance through enzymatic production was also recorded. Aminglycoside-acetyltransferases AAC(3)-I and AAC(6’)-I phenotypes were found respectively in 16/36 (44.4%) and 20/36 (55.6%) of aminoglycosides resistant isolates, thereby explaining the resistance phenotypes observed with gentamycin and amikacin. On the other hand, phenotypes associated with quinolones resistant isolates were produced through natural resistance and acquired resistance through non enzymatic drug resistance mechanisms. Resistance of isolates to quinolones was mainly due to an association of target modification (gyrA and parC), porin reduction and/or efflux mechanism which was present in 107/213 (49%) of quinolones resistant isolates, wild type phenotype was found in 60/213 (28%) of quinolone resistant isolates (Table 3).

**Table 3:** Resistance mechanism/phenotype of resistant isolates

Resistance mechanism/phenotype		N° of isolates (%)
To betalactamines	ESBLs	68/105 (64.7)
	Cephalosporinases	27/105 (25.7)
	Carbapenemases	7/105 (6.7)
	Penicillinases	3/105 (2.8)
To aminoglycosides	AAC (6’)-I	20/36 (55.6)
	AAC (3)-I	16/36 (44.4)
To quinolones	gyr A + parC + porin reduction and/or efflux	107/213 (49)
	Wild type	60/213 (28)
	gyrA	32/213 (15)
	Porin reduction	8/213 (4)
	gyrA + parC	6/213 (3)
	Efflux	3/213 (1)

## Discussion

Knowledge of the etiology and the antimicrobial resistance patterns of the agents involved in UTIs may help clinicians to choose appropriate antimicrobial treatments. The present study determined the bacteriological profile and antimicrobial susceptibility patterns of urine culture isolates among patients in Ndjamena General Hospital of National Reference, Chad. In this study, 216 (32.7%) on 660 urine samples met the criteria for urinary tract infection. This data contrasts with those presented by other surveillance studies. Irenge *et al.* [8] among 2,724 urine samples processed in a tertiary care hospital in south Kivu Province (Democratic republic of Congo) found a rate of 23.6%. Beyene and Tsegaye [9] obtained a bacteriuria of 9.2% from 228 cultured urine specimens in Jimma University Specialized Hospital in Southwest Ethiopia whereas Villar *et al.* [10] recorded a result of 25.5% from 3.105 urine samples collected from male outpatients in Argentina. The data reported in this study showed a high bacteriuria in inpatients compared to outpatients. This result is in line with that of Gupta *et al.* [11] and might reflect infections acquired during hospitalization. *E. coli* is the major etiological agent involved in urinary tract infections accounting for up to 90% of cases [5]. *E. coli* was the most common bacteria isolated from urine samples in both inpatients and outpatients of both sexes and this finding is in agreement with other studies [1,12-14]. In contrary to another study where the second reported isolate was *Proteus mirabilis* [15], in this study, it was *K. pneumonia* followed by *E. cloacae* which is in agreement with the findings of Ghorbani *et al.* [1].

Resistance to antimicrobial agents has been noted since the first use of these agents and is an increasing worldwide problem [16]. The Infectious Diseases Society of America guidelines suggest that 10-20% resistance warrants a change in the recommended antibiotic used as the first line therapy [17]. In the present study, only imipeneme (94.9%) displayed satisfactory activity against pathogens. High antibiotic-resistance rate was observed with other antimicrobial drugs. Resistance of isolates to betalactamines was mainly due to production of ESBLs. It has been found that the increasing frequency of ESBL phenotypes is an emerging problem due to the enormous potential for multidrug resistance from strains that produce these enzymes [18]. Acetylation of aminoglycosides by acetyltransferases is one of the major mechanisms of acquired resistance to these compounds [19, 20]. The 3-*N*-aminoglycoside acetyltransferases (AAC(3) enzymes) and the 6’-*N*- aminoglycoside acetyltransferases (AAC(6’) enzymes) were found to be among the modifying enzymes most commonly encountered in clinical isolates [19, 21]. The AAC(3)-I enzymes confer resistance to gentamycin, sisomicin and astromicin and are widespread among Enterobacteriaceae [21, 22]. These findings were also observed in the current study. The involved mechanisms of resistance to quinolones were alterations in the targets of quinolones, decreased accumulation due to impermeability of the membrane and/or an over expression of efflux pump systems and an association of many of these. This result agrees in some respect with other studies [23, 24].

The decreased susceptibility rates found for many agents in the current study is worrisome, since some of them are currently prescribed in Ndjamena as first line agents for treating hospital or community acquired infections such as UTIs. The current data may reflect the extensive use of prescribed agents. This over use may select for multidrug resistant strains, harboring the potential to disseminate within a specific region.

## Conclusion

*E. coli* remains the most common bacterial uropathogen responsible for UTIs in Ndjamena. This study confirms the presence of antibiotic-resistant uropathogens in this study area. As drug resistance is an evolving process, routine surveillance and monitoring studies should be conducted to provide physicians with knowledge about the most effective empirical treatment of UTIs.

### What is known about this topic

- *E. coli* is the most important cause of urinary tract infections;
- Antibiotics resistance of uropathogens varies from countries and regions;
- Infections caused by resistant microorganisms often fail to respond to empiric treatment.

### What this study adds

- *E. coli* is the most common bacterial uropathogen responsible for UTIs in Ndjamena;
- Imipenem is the appropriate antibiotic for treating UTIs in Ndjamena;
- Antibiotics resistance of the uropathogen responsible for UTIs occurs mainly through production of extended spectrum betalactamases (ESBLs).

## Competing interests

The authors declare no competing interests.
